# Key role of excess atomic volume in structural rearrangements at the front of moving partial dislocations in copper nanocrystals

**DOI:** 10.1038/s41598-019-40409-9

**Published:** 2019-03-07

**Authors:** S. G. Psakhie, K. P. Zolnikov, D. S. Kryzhevich, A. V. Korchuganov

**Affiliations:** Institute of Strength Physics and Materials Science SB RAS, 634055 Tomsk, Russia

## Abstract

Here we report on a molecular dynamics simulation of the atomic volume distribution in fcc copper with moving partial dislocations 1/6 〈112〉 {111}. The simulation shows that the leading and trailing partial dislocations surrounding a stacking fault move via local fcc→hcp and hcp→fcc transformations and that a fcc–hcp transition zone exists in which the atomic volume is larger than that in the perfect close-packed structure. The excess volume is five to seven percent, which compares with volume jumps on melting. The simulation results agree with experimental data showing that the nucleation of dislocations is preceded by the formation of regions with an excess atomic volume.

## Introduction

Amorphous and crystalline materials are essentially different in atomic structure, and plastic deformation in them nucleates and evolves differently. The plastic behavior of amorphous materials is considered in the context of shear transformation zones (STZs)^1–3^ whose nucleation and evolution in a material are a thermally activated process related directly to fluctuations of its free volume^4^. Both experiments and simulations show that the plastic shear in amorphous materials occurs in regions with an excess atomic volume (liquid zones) as possible STZ nuclei^5–8^, and this is because the energy barriers in such regions decrease and a local mechanical instability (softening) arises there^9^.

Unlike amorphous materials, crystalline materials are considered to develop plasticity via local structural transformations which provide the nucleation and motion of defects in their structure^10,11^, and the role of excess volume in the processes, as a rule, is left untouched.

It is known that the atomic density (atomic volume) is a thermodynamic variable which determines the phase state of condensed matter^12^. The phase diagrams plotted in terms of temperature, concentration, and atomic density not only extend the knowledge of phase states in a solid under changes of its atomic density but can also explain the instability and mechanical melting of its lattice when the solid expands to a critical specific volume^13^. Molecular dynamics studies suggest that the lattice instability owes to an increase in atomic volume^14^, no matter whether the increase is by heating, mechanical action, or buildup of defect concentrations, e.g., interstitial concentration^15,16^. For example, the fact that it is the increase in atomic volume which drives the lattice instability in a mechanically melted fcc material was established elsewhere^17,18^ and was confirmed by simulation data on the behavior of fcc copper with point defects^19^.

In the papers cited above^12–19^, the increase in volume was taken uniform. In this context, the questions arise of what effects can be produced by a local volume change and whether it will precede the formation of lattice defects, and if so, what types of these defects are. In mechanically loaded fcc metals, specific local transformations corresponding to fcc–hcp transitions were observed^20,21^. Such local transformations were considered as certain protodefects responsible for classical structural defects like partial dislocations, stacking faults, etc., and their mechanisms were studied^20,21^.

In this connection, it is of interest to investigate how the excess volume contributes to the incipient plasticity in crystalline materials and how this contribution correlates with that in amorphous materials.

The space-time scale of nucleation and evolution of local structural transformations is very small, and computer simulations are the best way for research in the processes^12,14^. For a more detailed study, it is expedient to simulate the nanoindentation of single crystals by the molecular dynamics method.

Here we report on a molecular dynamics simulation to clarify how the excess atomic volume in fcc metal contributes to the nucleation of local structural changes and how it provides the motion of partial dislocations.

## Results and Discussion

The simulation shows that the indenter–crystallite contact zone is the first region involved in local structural transformations. The transformations, as it follows from their analysis, are completely the same as those found elsewhere^20^, and they are due to specific rearrangements on the first and second coordination spheres of a center atom, whose immediate surrounding is thus involved in fcc→hcp transformation, and their nucleation is of thermofluctuation nature^21^.

**Figure 1:**
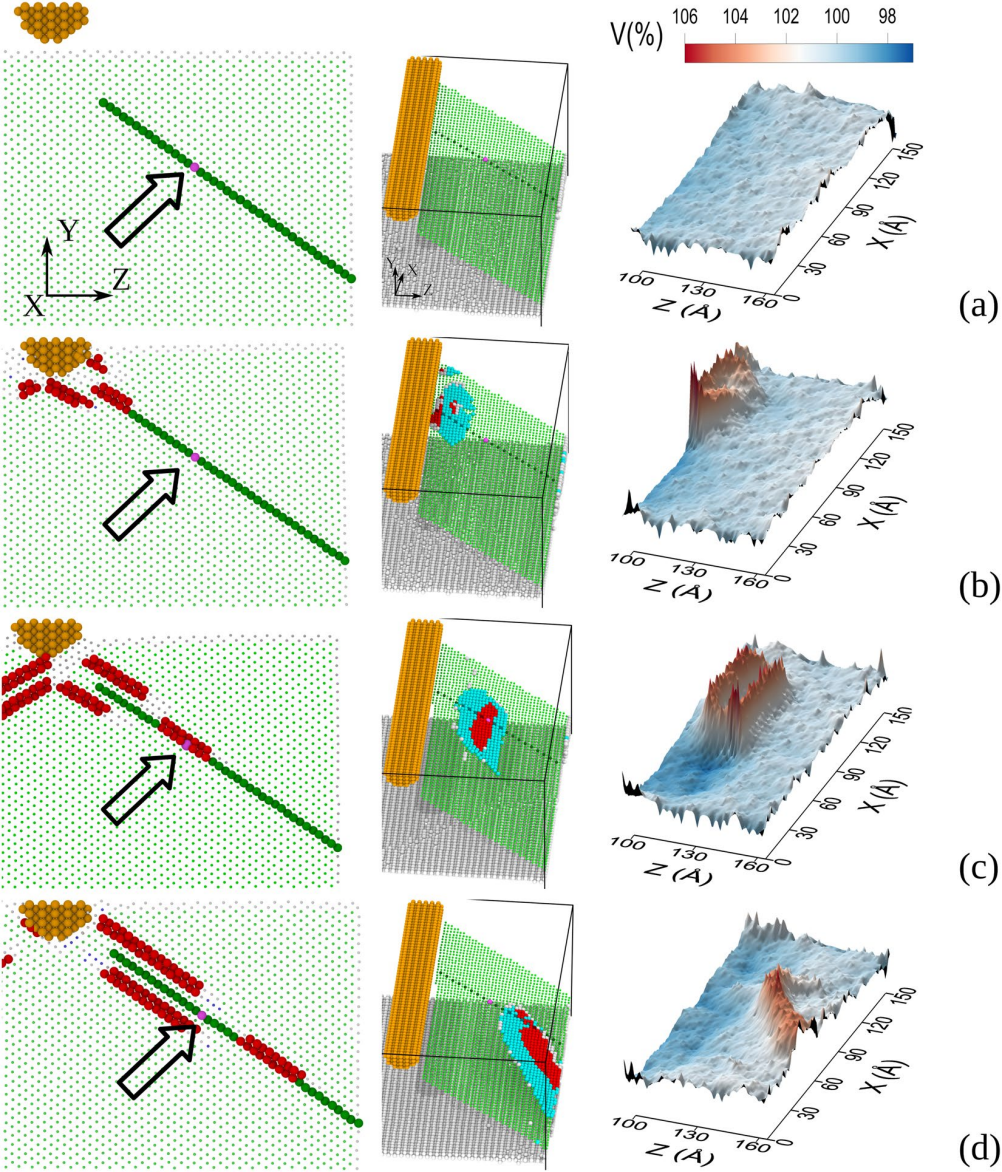
Structural fragment projected onto the (110) plane (left column), slip plane structure with a stacking fault (central column), and atomic volume distribution in the slip plane (right column) at different points in time: 3 ps (**a**), 5 ps (**b**), 9 ps (**c**), and 14 ps (**d**). Denoted by colors are atoms with fcc (green), hcp (red), and uncertain symmetry of immediate surroundings (gray), and atoms with increased local volume (blue).

Such local structural transformations in the model crystallite can be analyzed by tracing the evolution of its defect system in the (011) and (111) atomic planes. Figure 1 shows typical transformations in the (011) and (111) planes (left and central columns, respectively), and also typical atomic volume distributions in the (111) plane (right column).

As can be seen in the figure, the initial crystallite structure is free of defects (Fig. 1a). The interaction of the indenter and the crystallite surface gives rise to planar defects whose evolution involves a deviation of the atomic volume from its equilibrium value (Fig. 1b–d). Analysis of the alternating planes in such defect regions shows that intrinsic and extrinsic stacking faults are formed by center atoms (Fig. 1, left column). These center atoms (hereinafter, red spheres in the figures) can thus be considered as elementary plastic strain carriers. The stacking faults are surrounded by partial dislocations moving along {111} slip planes from the loading region to the free surface.

Let us analyze the dynamics of volume changes in the model crystallite by analyzing the state of atoms in one of its planes with a stacking fault (Fig. 1, left and central columns) which contains atomic chain *C* (dark green) with atom *A* (colored purple and arrowed) as a reference atom whose local surroundings undergo fcc→hcp and hcp→fcc transformations.

The motion of partial dislocations in this slip plane can be identified from the positions of a stacking fault in the left column of Fig. 1; its position in Fig. 1b is near the indenter. As the crystallite is loaded, the stacking fault changes the position, capturing atom *A* at an indentation depth of 5.0Å (Fig. 1c), and this suggests that an hcp transformation occurs in the immediate surroundings of atom *A*. The further motion of the partial dislocations surrounding the stacking fault brings it closer to the free surface, and at an indenter penetration depth of 6.0Å, the immediate surroundings of reference atom *A* transform from hcp to fcc (Fig. 1d).

As can be seen from Fig. 1, an increase in the local volume and structural transformations occur at the staking fault front in the (111) plane while the atomic volume behind the front decreases almost to its initial value. Thus, the stacking fault region is surrounded by atoms featuring a high atomic volume, and the excess volume can reach 5–7%. When the stacking fault reaches the free surface, it forms a jog whose size is determined by the number of partial dislocations appearing at the free surface.

**Figure 2:**
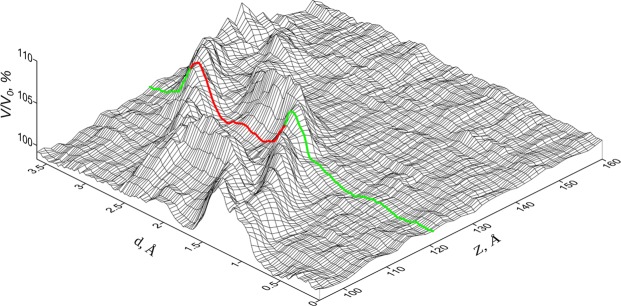
Spatial-temporal distribution of the relative volume for atomic chain *C*: *d* – indentation depth; *Z* – coordinates of atoms in chain *C* along the direction [100].

Figure 2 shows the spatial-temporal distribution of the relative volume calculated by (1) for atomic chain *C* in the [100] direction with the indication of indentation depths (*d*) and coordinates of atoms (*Z*). As can be seen, there are two clearly defined “ridges” corresponding to high values of the relative volume and a “valley” between them. Note that the structure of the immediate surroundings is hcp for atoms in the valley and fcc for atoms outside of the ridge system. This is illustrated in the figure by a two-color line (red for hcp, green for fcc) which demonstrates how the relative volume per reference atom changes with indentation depth.

It is also seen from Fig. 2 that at small indentation depths, the relative volume per reference atom changes by no more than 0.5%, and the change is due to thermal oscillations of atoms. A characteristic projection of the crystallite structure for small indentation depths is shown in Fig. 1a. As the indentation depth reaches *d* = 1.4Å, the relative volume per reference atom increases steeply such that the immediate surroundings of the atom transform from fcc to hcp structure. The transformation causes the motion of the leading partial which forms the stacking fault in the {111} slip plane. This result agrees with data on the possibility of phase transitions in metals and alloys with excess atomic volume^22^. The above local structural transformation can be considered as an elementary (single) event of the stacking fault motion in the plane (111). The stacking fault shifts its position toward the free surface by ~0.7 of the lattice parameter, and on completion of the structural transformation, the relative volume per reference atom decreases. It should be noted that the local volume for the region after direct fcc→hcp transition is somewhat larger (∼1%) than the equilibrium volume in fcc regions of the copper crystallite due to the effect of the surroundings. Thus, the reference atom becomes part of the staking fault, as illustrated in Fig. 1b.

At an indentation depth of 2.9Å, the surroundings of the reference atom start to experience a reverse hcp→fcc transformation which is also accompanied by an abrupt increase in the relative volume and its further decrease to the initial value. The transformation causes the motion of the trailing partial which recovers the fcc structure. A characteristic projection of the crystallite structure after reverse transformation is shown in Fig. 1c.

Our research data on the role of excess atomic volume in incipient plasticity agree with available experimental data. For example, according to *in*-*situ* high resolution TEM studies by Zheng and colleagues^23^, the nucleation of a partial dislocation in a tensile gold nanocrystal is preceded by a local increase of 5% in interatomic distances. As has been shown on the example of vanadium in one of the molecular dynamics simulations^15^, such an increase in volume is required for homogeneous melting in metals with a perfect bcc lattice and with point defects. These data agree well with the Born criterion according to which the expansion of a solid to more than a certain critical volume is impossible. On approaching this critical volume, e.g., by mechanical expansion, heating, addition of point defects, the crystallite suffers a “rigidity catastrophe” due to a jump-like decrease in its shear moduli^19^. In our calculations, the increase in the atomic volume at the stacking fault front compares with that on melting, measuring 5–7%.

The results of simulation for different loading rates and temperatures reveal the following features of structural rearrangements at the front of partial dislocations. Under loading with a rate of 5, 25, and 50 m/s at *Т* = 300 К, the average atomic volume at the dislocation front is 104.2, 104.0, and 103.8%, respectively. The slight decrease in the atomic volume is likely due to a delay of accommodation processes with respect to structural rearrangements induced by the indenter. Increasing the loading rate increases the delay, and this not only decreases the excess volume but it also increases the elastic limit^21^. According to the simulation, the atomic volume at a loading rate of 25 m/s remains the same irrespective of whether the temperature is 200, 300, or 400 К.

For quantitative assessment, the features of incipient plasticity are often considered in terms of activation volume^24–26^. Unlike the atomic volume, the activation volume is explicitly dependent on the crystallite temperature^27^. Calculations of the activation volume give its value equal to ~30*b*^3^, where *b* is the Burgers vector of a full dislocation 1/2 〈112〉 {111}. Results obtained are consistent with theoretical and molecular dynamics simulation data^24–27^.

## Conclusion

In summary, our molecular dynamics study of mechanically loaded copper shows that the use of an extended indenter in simulations allows one to generate isolated stacking faults and to trace their motion in model crystallites. This opens the way for detailed analysis of local fcc→hcp and hcp→fcc transformations which provide the motion of stacking faults along slip planes {111}. Our study is the first to demonstrate the existence of a transition zone which borders this type of defects and always features an excess atomic volume responsible for local lattice instability. The local increase in volume is a necessary component for the motion of both leading and trailing partial dislocations surrounding a stacking fault in copper. Our research data on local volume changes in defect nucleation regions agree with experimental results showing that the nucleation of partial dislocations in fcc nanocrystals is preceded by the formation of an excess atomic volume. The research data on the behavior of copper in the transition zone extends our knowledge about the role of excess volume in phase transitions^22^ and in the motion of stacking faults and partial dislocations in fcc metals. Reasoning from the study presented, we can state that it is the local excess volume which plays the key role in the nucleation and evolution of plastic deformation in both amorphous and fcc crystalline materials. In amorphous materials, the excess volume precedes structural transformations responsible for STZs, and in fcc crystalline materials, it precedes structural transformations responsible for partial dislocations as one of the main mechanisms of plasticity.

## Materials and Methods

**Figure 3:**
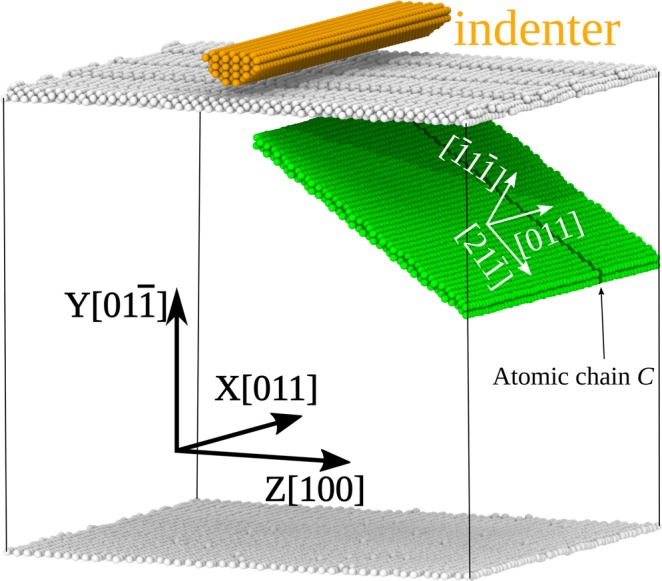
Initial structure and crystallographic orientation of the model system.

The model material was a fcc copper crystallite shaped as a cube with edges of size 160Å. The crystallite contained about 400 thousand atoms. It was thermally equilibrated to 300 K in an NVT ensemble using a Nose–Hoover thermostat. The model crystallite and its crystallographic orientations are shown in Fig. 3. The loaded face of the crystallite and its faces perpendicular to the crystallographic direction [100] were free, and the direction [011] was assigned periodic boundary conditions. For simplicity of our analysis and clarity of data representation, the crystallite was loaded with an extended semi-cylindrical indenter^28–30^. The indenter axis was oriented along the direction [011]; the load was applied to the middle of the crystallite face (01 $\bar{1}$). The indenter velocity in the direction [01 $\bar{1}$] was 25 m/s. The indenter radius was 10А, allowing us to locally act on the specimen and to obtain an isolated stacking fault. For the crystallite to be immobile as a whole, special boundary conditions prohibited its three lower planes (bottom layers) from shifting in the loading direction.

The interatomic interaction was described by a many-body potential constructed in the framework of the embedded atom method^31^. The potential allows one to very accurately describe the surface properties, structural defect energy, elastic parameters, and other characteristics of a solid which are important for simulating its structural response to mechanical loading.

The excess volume per atom was calculated as

1 $${\rm{\Delta }}{V}_{i}={(\frac{{\sum }_{j=1}^{{N}_{s}}|\overline{{r}_{i}}-\overline{{r}_{j}}|}{{N}_{s}})}^{3}/{V}_{i}^{0},$$

where ${V}_{i}^{0}$ is the equilibrium atomic volume; *N*_*s*_ is the number of nearest neighbors of the atom *i*; ${\bar{r}}_{i}$ and ${\bar{r}}_{j}$ are the respective radius vectors. The atoms corresponding to the centers of local structural transformations were determined on the basis of common neighbor analysis^32^. These atoms are further referred to as center atoms. The resulting structure was visualized in the OVITO software^33^. Simulations were carried out using the LAMMPS molecular dynamics code^34^ with a time step of 0.5 fs. The Velocity Verlet algorithm was used to integrate the equations of motion.

The activation volume was determined from the dislocation nucleation rate *J* calculated for different indentation depths. The dislocation nucleation rate is an important kinetic characteristic of plastic deformation and means the average number of dislocations formed in a crystallite per atom per unit time:

2 $${J}={1}/({N}\langle t\rangle ),$$

where *N* is the number of atoms in the deformed crystallite region beneath the indenter, <*t*> is the average expectation time for dislocation nucleation.

The data on the dislocation nucleation rate under mechanical load are often interpreted using the expression (see, e.g.^25,35^):

3 $$J={J}_{0}\,\exp \,(\frac{-U+{{\rm{\tau }}V}_{act}}{kT}),$$

where J_0_ is the maximum nucleation rate, *U* is the activation energy, *τ* is the shear stress in the slip plane of a dislocation, *k* is Boltzmann’s constant, *T* is temperature, *V*_*act*_ is the activation volume. Formulae (2) and (3) are used to approximate the results of simulation and to determine the activation volume.

For the discrete indenter structure not to affect the estimation accuracy of the dislocation nucleation time, the indenter represented an external cylindrical repulsion field acting on crystallite atoms with the force:

$$F(r)=-\,K{(r-R)}^{2}\,{\rm{for}}\,{r} < R,$$

$$F(r)=0\,{\rm{for}}\,r\ge R,$$

where *K* = 10 eV/Å^3^ is a constant, *r* is the distance to the indenter axis, *R* = 10Å is the indenter radius. The use of the field indenter excludes the “jump-to-contact” effect due to the attraction of surface atoms to the indenter at distances corresponding to the attracting branch of the interaction potential. Note that the character of structural transformations at the front of partial dislocations is the same for the field and discrete indenters.

## Data Availability

The raw/processed data required to reproduce these findings cannot be shared at this time as the data also form part of an ongoing study, but feel free to contact the corresponding author at sp@ispms.ru to get them.
